# Supportive needs of women who have experienced pregnancy termination due to fetal abnormalities: a qualitative study from the perspective of women, men and healthcare providers in Iran

**DOI:** 10.1186/s12889-019-6851-9

**Published:** 2019-05-03

**Authors:** Bahareh Kamranpour, Mahnaz Noroozi, Massoud Bahrami

**Affiliations:** 1 Student Research Committee, School of Nursing and Midwifery, Isfahan University of Medical Sciences, Isfahan, Iran; 2 Department of Midwifery, College of Nursing and Midwifery, Rasht Branch, Islamic Azad University, Rasht, Iran; 3 Department of Midwifery and Reproductive Health, School of Nursing and Midwifery, Isfahan University of Medical Sciences, Isfahan, Iran; 4 Department of Adult Health Nursing, School of Nursing and Midwifery, Isfahan University of Medical Sciences, Isfahan, Iran

**Keywords:** Supportive needs, Therapeutic abortion, Congenital abnormalities, Birth defects, Qualitative study, Iran

## Abstract

**Background:**

Extensive application of screening tests for early diagnosis of fetal abnormalities would justify support for women who are facing pregnancy termination due to fetal abnormalities. Considering the lack of available information regarding supportive sources for these people, the present study was conducted to determine the supportive needs of women who have experienced pregnancy termination due to fetal abnormalities.

**Methods:**

The present research was a qualitative study. The participants were selected using a purposeful sampling method with maximum variation. Data were collected through in-depth personal interviews and taking of field notes and were analyzed simultaneously using conventional content analysis.

**Results:**

The main categories that appeared in the present study included “support from the husband” with sub-categories of “mental support and necessary accompaniments”, “participating in planning for future pregnancy” and “financial support to pay the costs of diagnosis and follow-up”, “support from the family and friends” with sub-categories of “helping in taking care of other children”, “help in performing daily activities” and “empathy, companionship and necessary support to maintain mental peace” and finally “support from peers” with sub-categories of “communicating with the peers and receiving information from them” and “creating a sense of confidence and hopefulness”.

**Conclusions:**

Results of the present study, by determining and highlighting the supportive needs of women who have experienced pregnancy termination due to fetal abnormalities, could be an appropriate basis for providing effective strategies to improve constant participation of the husbands, family members and the peers along with other professional care.

**Electronic supplementary material:**

The online version of this article (10.1186/s12889-019-6851-9) contains supplementary material, which is available to authorized users.

## Background

The experience of pregnancy is usually considered a desirable transition in women’s lives and is associated with unique physical and mental changes. This transition changes women’s image of themselves by establishing a sense of commitment due to the birth of the neonate and would make them hopeful of a healthy child [1]. Diagnosis of fetal abnormalities is considered an unexpected incident for the mother and the family and is associated with severe emotional trauma. This issue would present women with various challenges including continuing the pregnancy without any interventions or elective termination of the pregnancy [2]. Elective termination in a wanted pregnancy, at any gestational age, is considered as loss of a child and is characterized by loss of hope for the future, expectations and the role of parenthood [3]. Considering that, at the time of diagnosis of fetal abnormalities, definitive prediction of the results in the range of non-fatal to fatal is not possible; decisions made in this situation are emotionally challenging [4]. Furthermore, in comparison to other similar situations, less social support exists for these situations because in the existing cultural context women would be blamed for deciding to terminate the pregnancy and having a fetus with abnormalities; so they could not easily share their decision with others. This would make them more lonely and vulnerable [5]. Results of the study by Rapp also showed that failure to have a healthy child, fear of being responsible for the abnormalities and attachment to the fetus would cause severe grief in women [6]. On the other hand, women’s active role in ending the fetus’ life is a unique circumstance that would distinguish its grieving process from the grief of spontaneous abortion or fetal death [1]. This action followed with several psychological consequences including intense mourning, feeling of guilt and humiliation, low self-esteem, anxious reactions, fury, doubt about the correctness of the decision, and fear of social judgment [2]. Nevertheless, the work of Korenromp and colleagues showed that while distress is very real, regret is rare [7]. Various studies have reported evidence of these reactions especially during the early weeks and months after terminating the pregnancy [8]. Results of the study by Maguire et al. showed that 17% of women would experience the symptoms of post-traumatic stress disorder (PTSD) until 2 to 7 years after terminating the pregnancy [9]. However, the existing studies have shown that these women would not receive sufficient support. Due to lack of information about this kind of loss, they could not even mourn the loss of their child [5]. The increasing growth in application of screening tests would justify the increasing need for providing necessary support for people with diagnosed abnormalities in their fetus. Although the emotional and psychological outcomes of pregnancy termination due to fetal abnormalities have been investigated in various studies, few studies have been conducted about the appropriate support for these people after losing the fetus. Although currently in some countries online groups are working to support women who have experienced pregnancy termination due to fetal abnormalities, it is still not determined whether this kind of support is adequate for their needs and desires [1].

Currently, in our country, fetal health screening tests are performed routinely and for all pregnant women who refer to public and private centers for prenatal care to avoid abnormal childbirth. Therapeutic abortion is legal with the diagnosis of three obstetrician and forensic medicine confirmation for fatal abnormalities causing severe maternal suffering, before the fourth month of pregnancy (or before the 19th week of pregnancy) based on authorized periodical ultrasonography with the consent of the woman. Although screening tests are performed as part of routine care for all mothers, these tests are not free, and the people will pay part of the cost depending on the type of health insurance that they are covered. Also, people who are not covered by the insurance will incur a lot of expense for these tests.

It seems that despite screening tests as part of routine care during pregnancy in the country and considering the issue of therapeutic abortion in cases of fatal abnormalities, there is still no codified program that meets the specific needs and problems of these individuals. Also, despite the physical, emotional and psychological outcomes of pregnancy termination due to fetal abnormalities in women, this group is still being ignored by the care systems and has been neglected by researchers and policy makers. Considering the different aspects of experiencing pregnancy termination due to fetal abnormalities, determining the supportive needs of these women could provide an appropriate context for systematic, comprehensive, cultural-based interventions and caring programs in the society. Since qualitative research is an approach for discovering and describing individual’s experiences and giving meaning and understanding to them [10], the present qualitative study was conducted to determine the supportive needs of women who have experienced pregnancy termination due to fetal abnormalities.

## Methods

The present qualitative research used a content analysis approach.

### Settings, samples and recruitment

In the present study, participants were 27 women who had experienced pregnancy termination due to fetal abnormalities and been referred to the health centers of Rasht, Iran, along with two men whose wives had experienced pregnancy termination due to fetal abnormalities and 13 healthcare providers (three midwives, one nurse, four gynaecologists, two forensic medicine specialists, two reproductive health specialists and one psychologist). Eligible participants were accessed through maternity wards of hospitals, prenatal clinics and offices of midwives, gynaecologists, and forensic medicine specialists. They were recruited directly or telephone numbers were obtained and they were subsequently telephoned. Samples were selected using a purposeful sampling method. The inclusion criteria were willingness to participate in the study and giving informed consent, being able to understand the questions and being able to read and write, having passed at least 1 year from the pregnancy termination experience and lack of any diagnosed psychological disorders. Demographic characteristics of the 42 participants are shown in Tables 1 and 2.

**Table 1:** Demographic characteristics of women who have experienced pregnancy termination due to fetal abnormalities and men

Age (years)	20–40
Educational level	Middle school degree (3), Diploma (9),
	Associate’s degree (2), Bachelor’s degree (13)
	Master’s degree and Ph.D. (2)
Occupation	Employee (9), Housewife (7), Laborer (11), Freelancer (2)
Number of children	No children (15),1 to 2 children (14)

**Table 2:** Demographic characteristics of healthcare providers

Age (years)	30–60
Working experience (years)	1–30
Field of expertise	Forensic medicine (2), Obstetrics and gynecology (4), Psychology (1) Reproductive health (2), Midwifery (3), Nursing (1)

### Data collection

Data were collected using in-depth semi-structured personal interviews and recording of field notes between October 2017 and April 2018. In this research, interviews were conducted with 42 participants. Interviews with five participants were conducted in two sessions, resulting in 47 interviews. After reaching eligible participants, none of them refused to participate in the study.

The first author (BK), who has 12 years working experience in midwifery, conducted the interviews. Other authors have previous interviewing experience and qualitative paper/report writing. Prior to data collection, the first author wrote down initial preconceptions and beliefs about the research topic based on her previous working experience and from a review of the literature. The interviews commenced with the general question of “Please explain your feelings when you learned about your fetus’s abnormality and you decided to terminate the pregnancy? What needs did you have afterwards?” and then were guided by the open and interpretative answers of the participants. The interviews of other participants commenced with the general question of “What do you think are the problems and needs of women who have experienced pregnancy termination due to fetal abnormalities?”(See Additional files 1, 2 and 3 for copies of the topic guides). The interviews lasted between 25 and 100 min and continued until there was data saturation and no new data code emerged in the interviews. The places and time for the interviews were selected based on the participants’ preferences.

### Data analysis

Data were analyzed using conventional content analysis [10]. Data analysis was performed simultaneously with data collection. All interviews were digitally recorded. The interviews were transcribed verbatim by the first author (BK). The interviews were then reviewed repeatedly to gain a comprehensive overview of the content. The sentences and phrases were then coded and, after inductive coding, similar codes were merged and those with similar meanings were put in the same groups and formed the sub-categories. Then, after comparing the sub-categories with each other, groups with similar concepts were put in the same main category.

### Rigor and trustworthiness

To maintain the rigor of the data, various methods including in-depth interviews at different places and times, combining different methods of data collection such as personal interviews and field noting, and selecting the participants with maximum variation (regarding their educational level, socio-economic status, occupation, age, number of children and the duration passed from the pregnancy termination) were applied. In this study, transcripts were returned to participants for comments or corrections. Also, to confirm the validity of the data, during other sessions, the coded interviews were given to four of the participants and their final opinions were achieved; so that member check could be obtained. The opinions of four experts were also obtained to assure the consistency of the results. In the present study, to enhance the transferability of the data, results of the study were given to three individuals with similar characteristics to the participants who did not participate in the study to judge the similarity of the results to their own experiences.

### Ethical considerations

The present study was approved by the Ethics Committee of Isfahan University of Medical Sciences (ethics code: IR.MUI.Rec.1395.3.945). The reasons for the study were explained prior to each individual interview. Also, obtaining informed consent, maintaining anonymity, confidentiality of the data and the right of withdrawal at any desired time were practiced.

## Results

During data analysis, 66 codes and eight sub-categories were developed. The final three main categories were “support from the husband”, “support from the family and friends” and “support from the peers” (Table 3).

**Table 3:** Results of data analysis

Code	Sub-category	Main category
*Consolation and empathy with the wife *Giving hope to the wife *Supporting the wife against others *Being present and accompanying the wife in the labor room if possible *Supporting the wife for returning to work	Mental support and necessary accompaniment	Support from the husband
*Reaching mutual understanding about the next pregnancy and its time *Helping in physical and mental preparation for the next pregnancy *Accompanying the wife for performing required care and measures for the next pregnancy	Participation in planning for future pregnancy	
*Providing financial support for screening and diagnostic tests *Providing financial support for genetic consultation *Providing financial support for counseling with a psychiatric or a psychologist	Financial support to pay the costs of diagnosis and follow- up	
*Caring for and keeping other children *Providing emotional support for other children	Helping in taking care of other children	Support from the family and friends
*Providing help in performing daily household activities *Preparing the house before wife’s discharge from the hospital	Helping in performing daily activities	
*Receiving empathy and understanding the emotional situation from the relatives *Helping in resolving women’s concerns and anxiety *Accompanying the woman for performing diagnostic and therapeutic measures	Empathy, companionship and necessary supports to maintain mental peace	
*Establishing communication with the peers for accepting the situation *Being informed about the process of pre-pregnancy consultations *Being informed about the process of prenatal care *Being informed about the appropriate manner for confronting the husband and the relatives	Communicating with the peers and receiving information from them	
*Having hope for life *Encouraging the mother for returning to normal life *Decreasing women’s concerns and fears about the recurrence of the incident *Gaining more energy through visiting the peers	Creating a sense of confidence and hopefulness	Support from the peers

### Support from the husband

Participants believed that husbands, as the closest person to women, had an important role in providing comprehensive support for women. Therefore, if they could understand women’s mental condition, empathies with them and show acceptance of the subject in their behavior and performance, women could overcome this difficult situation faster and the hope of having a healthy child in the next pregnancy would be strengthened for them. This main category is consisted of three sub-categories.

#### Mental support and necessary accompaniments

Most of the participants mentioned that the husband has a key role in providing support for women in accepting and dealing with the incident and returning to normal life. They believed that empathy with the wife at the time of hearing the news about fetal abnormality and during the process of decision-making for terminating the pregnancy, full-time presence of the husband in the hospital and in the labor room, if possible, giving hope to the wife for the future, making the effort to realize the wife’s concerns and supporting her in cases such as others’ misconception about the reason for the incident, by the husband, are effective in creating mental peace for the wife after losing the fetus.

*“I knew that my husband was upset but he was not showing his discomfort around me, he supported and comforted me, I really did not know what to do if he was not around … he always had my back.” (Woman).*

#### Participating in planning for future pregnancy

According to the participants, hope for having a healthy child in the next pregnancy would help a woman maintain her hope for life and her self-esteem. They emphasized the supportive role of the husband regarding understanding the issues of the next pregnancy and its timing, helping with physical and mental preparation for the next pregnancy, accompanying the woman for prenatal care and also performing genetic counseling, if necessary.

*“Well, I had to come to Rasht for every visit, test or ultrasound; although my husband works with his car but he would have brought me every single time. He did not accept that I would come and go alone, by myself.” (Woman).*

#### Financial support to pay the costs of diagnosis and follow-up

Based on the participants’ statements, supporting the wife in financial issues is another supportive act that would strengthen the basis of the married life and would provide mental peace for the woman. Providing the diagnostic and screening costs and also the costs of necessary counseling by the husband was the request of almost every participant.

*“My physician told me to go for genetic counseling before my next pregnancy and perform amniocentesis after getting pregnant. When I told my husband he said that choose one, how much should I spend?... I did not pursue it because he was not consent.” (Woman).*

### Support from the family and friends

Participants stated that receiving support from the family, friends and close relatives would be effective in finding mental peace, decreasing stress and anxiety and helping with the daily duties and activities. This main category is consisted of three sub-categories.

#### Helping in taking care of other children

Some of the participants narrated that during the diagnostic procedures and therapeutic abortion, during the period of hospitalization and even after discharge, if there was a lack of the necessary support from the family and friends for taking care of other children, the couple would encounter numerous concerns and problems, due to the bad psychological condition of the mother.

*“When I was coming to Rasht for taking the letter from the Forensic Medical center, I had no one to take care of my first child; because my father is dead, my mother has a heart condition and my husband’s family does not live here … I had to bring my child to the hospital and Forensic Medical center a few times, it was really hard.” (Woman).*

#### Helping in performing daily activities

Based on the participants’ statements, due to the inconvenient mental condition, during the process of treatment and even for a while after the pregnancy termination, some women were not able to maintain their house affairs and satisfy the needs of other family members and felt an urgent need for others’ support to maintain their household.

*“For a while, I could not do anything. If my mother and husband were not around I do not know who would have managed this house! I was not in the mood for looking after my first daughter’s homework … or cook her food that she likes.” (Woman).*

#### Empathy, companionship and necessary supports to maintain mental peace

Reaching peace is the main concern of many women who have experienced pregnancy termination due to fetal abnormalities. After accepting the truth, they were searching for a way to be effective in returning peace to their life by decreasing their mental pressures and controlling their stress. The women believed that empathy and companionship of the family and friends could be effective in maintaining peace and decreasing stress and anxiety. The participants emphasized the positive effect of relatives’ visits at different times to decrease the feeling of loneliness and drained emotions, pass the stages of mourning, decrease stressful factors and help with following up the treatment process after pregnancy termination.

*“I have a cousin I am very comfortable with, after terminating the pregnancy I only wanted her to be around me … whenever I had a problem or needed something I called her. She understands me. Whenever my husband was busy and could not come to the physician’s office with me, I would have called her and she never said no.” (Woman).*

### Support from the peers

According to the participants, visiting and interacting with similar women who have experienced pregnancy termination due to fetal anomalies and observing their success in passing this difficult period and having a successful pregnancy after this incident could facilitate acceptance ofand compatibility with this experience. This main category is consisted of two sub-categories.

#### Communicating with the peers and receiving information from them

Most of the participants believed that communicating with women with similar experiences would be beneficial and considered hearing about their experiences, receiving information about prenatal cares for the next pregnancy, performing genetic or psychological counseling, pregnancy care, and the manner of dealing with relatives and the husband, very informative. They mentioned that using peers’ experiences could be effective in helping to pass the stages of mourning and acceptance of this bitter experience.

*“When I spoke to one of our far relatives who have experienced the same problem, I realized so many things and even her words made me go to a psychologist and receive counseling … now I feel so much better … her experiences really helped me.” (Woman).*

#### Creating a sense of confidence and hopefulness

According to the participants, communicating with people with similar experiences who later had another child would make them better cope with this reality and would significantly decrease their concerns, anxiety and stress and would strengthen their hope for the future.

“*When a woman sees that someone could have a healthy child after a problematic pregnancy, it would decrease her anxiety, increase her hope for the future …*” *(Nurse).*

## Discussion

The present study was conducted to determine the supportive needs of women who have experienced pregnancy termination due to fetal abnormalities. Results of the present study showed that support from the husband, family, friends and peers are the most important sources of support for these women.

Although there are few studies about the supportive role of the husband after pregnancy termination due to fetal abnormalities, results of studies about other causes of fetal loss including intrauterine death and spontaneous abortion)pregnancy loss before the 20th week of gestation) showed that the quality of the couple’s relationship could be effective in their reaction toward this incident. In this regard, results of the study by Avelin showed that, although parents have different methods for mourning, most of the couples would be able to accept the issue and respect each other. On the other hand, most of the mothers are willing to express their feelings clearly after this incidence and share the experience of this loss with their husband [11]. In the study of Toller et al., women who had experienced loss of their fetus also believed that empathy from their husband and his affection toward them which is a sign of accepting this reality could be effective in decreasing their sense of fault and guilt [12]. Results of the study by Anderson et al. revealed that emotional support from the husband, along with professional support, is effective in resolving the concerns and adjustment with this incident [13].

Women participants in the present study also demanded the empathy and presence of their husbands at the time of finding out about the abnormality, while making the decision about terminating the pregnancy, at the time of hospitalization, when returning home and while planning for the next pregnancy. They believed that emotional support from the husband is effective in accepting and coping with the loss. Results of the present study showed that being hopeful of having a healthy child in the next pregnancy would be helpful in maintaining women’s hope for the future and self-esteem. If this desire would be ignored or neglected by the husband, women would suffer significant mental damages. In line with the results of the present study, results of the study by Asplin mentioned that predicting the time of the next pregnancy, especially in older women, is of great importance [14]. It was also determined in a study that lack of sufficient emotional support from the sexual partner could be a strong predictor of long-term undesirable psychological outcomes (post-traumatic stress) in women who have experienced pregnancy termination due to fetal abnormalities [7]. Also, in the study of Ramdaney, having financial capability was considered an effective factor in benefiting from supportive resources [15]. Therefore, husbands should be educated to be aware of women’s emotional needs after this incident and consequently assure their wives that no one blames them for this loss. Also, given that counseling services are not covered by insurance, insurance coverage of counseling and psychological services for women with experience of termination of pregnancy due to fetal abnormalities could be helpful in providing financial support by the husband for his wife to pay the relevant costs. Furthermore, health care providers can use this information to meet the supporting needs of women with a pregnancy termination due to fetal abnormalities in various ways by careful planning and good coordination.

Results of the present study showed that receiving emotional support from the family and close friends is effective in restoring mental peace and decreasing the anxiety and stress of women. In fact these women believed that family’s support is effective in providing empathy, and decreasing the feeling of guilt and self-blame. According to them, in this situation, cooperation of the family members and close friends could be a significant help in balancing the situation and returning to normal condition. Also, in the study of Anderson, presence of a friend or a relative was effective in resolving the concerns and adjustment with the loss [13]. Similar to the present study, in a study, most of the women expected to be supported emotionally by their family and friends and lack of sufficient support influenced mothers’ decisions about finding other supportive resources [15]. According to the results of previous studies, the quality of emotional support from the family and friends would change overtime and so these women would feel they have not been supported and have been left alone in their mourning. This decrease in the emotional support would make women search other support resources, although they did not expect it at the time of pregnancy termination [16]. Undoubtedly, presence of constant emotional support from the family and friends in accordance with the special condition of these people is effective in improving their psychological compatibility with the conditions of pregnancy termination due to fetal abnormalities.

Results of the present study showed that experiencing the loss of a fetus is a weakening experience for women, which is accompanied by lowered self-esteem and a sense of loneliness. Therefore, meeting people with similar experiences in the past and benefiting from their experiences for accepting the incident, showing care and receiving counseling and returning to normal life is effective. In a study, willingness to meet people with similar experience online and anonymously was also mentioned as an effective factor in making the decision about participating in supportive systems [15]. In this regard, Cacciatore et al. in their study showed that, in supportive groups, where people share their stories, the process of coping with the trauma would be formed. This relationship between two or more people with similar experiences would help the mournful person find a meaning to their experience [17]. In the study of Roehrs, mothers mentioned the difficulty of expressing their experience to their friends and family and indicated the benefits of support from the group of peers to accelerate reaching the acceptance stage [18]. Since, in these women, mourning for a child that was never born and they have no memory of is difficult, and also the existence of the abnormality has minimized their sorrow for others, these silent mourners need to communicate with those who have similar experiences to them more than ever [16, 19]. In this situation, women would accept information from their peers more easily and more openly discuss their issues and problems with them.

Generalization of the results of the present study should be performed cautiously due to the qualitative nature of this study. Although qualitative studies have no claim in generalizability of the results, they could be important for those who are willing to apply their results and consider it as a limitation. In this regard, the effort has been made to increase the accuracy and power of data transferability by selecting participants with maximum variety, and receiving guidance and supervision from experts and external reviewing.

## Conclusions

This study has presented the supportive needs of women who have experienced pregnancy termination due to fetal abnormalities from different aspects. It appears that, by determining and emphasizing on these needs and presenting them to the health system authorities, effective strategies for improving constant participation of the husband, family members and friends and also peers along with other professional care for these people could be provided and the conditions for their rapid return to normal life would be provided.

## Additional files

Additional file 1: Interview guide during the face-to-face interviews with women who have experienced pregnancy termination due to fetal abnormalities for the study conducted to determine the supportive needs of these women from the perspective of women, men and healthcare providers in Rasht Town, Iran, 2017–2018 (See methods section for further description). (DOCX 15 kb)

Additional file 2: Interview guide during the face-to-face interviews with men who their wife have experienced pregnancy termination due to fetal abnormalities for the study conducted to determine the supportive needs of these women from the perspective of women, men and healthcare providers in Rasht Town, Iran, 2017–2018 (See methods section for further description). (DOCX 15 kb)

Additional file 3: Interview guide during the face-to-face interviews with health care providers (midwives, nurses, gynaecologists, forensic medicine specialists, reproductive health specialists and psychologists) for the study conducted to determine the supportive needs of these women from the perspective of women, men and healthcare providers in Rasht Town, Iran, 2017–2018 (See methods section for further description). (DOCX 15 kb)

## Abbreviations

PTSD: Post-Traumatic Stress Disorder
