# Supplementary material for: Supportive needs of women who have experienced pregnancy termination due to fetal abnormalities: a qualitative study from the perspective of women, men and healthcare providers in Iran
Source: BMC Public Health. 2019 May 3;19:507. doi: 10.1186/s12889-019-6851-9 (PMC6500064; doi:10.1186/s12889-019-6851-9)
Supplement: Supplementary file 1 — Interview guide during the face-to-face interviews with women who have experienced pregnancy termination due to fetal abnormalities for the study conducted to determine the supportive needs of these women from the perspective of women, men and healthcare providers in Rasht Town, Iran, 2017–2018 (See methods section for further description). (DOCX 15 kb) [file 12889_2019_6851_MOESM1_ESM.docx]

**Additional file 1:** Interview guide during the face-to-face interviews with women who have experienced pregnancy termination due to fetal abnormalities for the study conducted to determine the supportive needs of these women from the perspective of women, men and healthcare providers in Rasht Town, Iran, 2017-2018 (See methods section for further description).

**Introduction:** *Aim, to create appropriate atmosphere*

- Name of the interviewer and affiliation
- Purpose of the study
- Consent to take part in the study
- Confidentiality, explain how the data will be used
- Interview will last approximately 30-60 minutes
- Audio recorded to ensure interviewer can fully engage in the interview

**Warm up questions:** *Aim\ make participants comfortable*

1. Please introduce yourself?

2. How old are you?

3. What is your education level?

4. What is your job?

5. How many pregnancies have you had?

6. How many children do you have?

7. How long is it since your pregnancy was terminated?

8. What was the gestational age of fetus during the termination of pregnancy? What was the problem?

9. Can I ask what happened to you after the diagnosis of fetal abnormalities?

**Interview guide questions in individual interviews with women who have experienced pregnancy termination due to fetal abnormalities**

1. After you found that your fetus had a severe abnormality and that you had to end your pregnancy, from whom did you expect to receive support? Please explain?

2- How do you think your husband would be able to support you in those circumstances? What would you like your husband to do for you?

3- Do you think it is necessary for your husband to be present at the time of the miscarriage of the fetus in the delivery department? Please explain?

4- How would you like your husband to help you after your discharge from the hospital?

5. In addition to your husband, from who would you expect more support?

6. How could your family and that of your husband support you? What would you like them to do for you?

7. Was there anyone other than your husband and family members whose support you think you needed? What help could they provide? Please explain?
