# Supplementary material for: Supportive needs of women who have experienced pregnancy termination due to fetal abnormalities: a qualitative study from the perspective of women, men and healthcare providers in Iran
Source: BMC Public Health. 2019 May 3;19:507. doi: 10.1186/s12889-019-6851-9 (PMC6500064; doi:10.1186/s12889-019-6851-9)
Supplement: Supplementary file 2 — Interview guide during the face-to-face interviews with men who their wife have experienced pregnancy termination due to fetal abnormalities for the study conducted to determine the supportive needs of these women from the perspective of women, men and healthcare providers in Rasht Town, Iran, 2017–2018 (See methods section for further description). (DOCX 15 kb) [file 12889_2019_6851_MOESM2_ESM.docx]

**Additional file 2:** Interview guide during the face-to-face interviews with men who their wife have experienced pregnancy termination due to fetal abnormalities for the study conducted to determine the supportive needs of these women from the perspective of women, men and healthcare providers in Rasht Town, Iran, 2017-2018 (See methods section for further description).

**Introduction:** *Aim, to create appropriate atmosphere*

- Name of the interviewer and affiliation
- Purpose of the study
- Consent to take part in the study
- Confidentiality, explain how the data will be used
- Interview will last approximately 30-60 minutes
- Audio recorded to ensure interviewer can fully engage in the interview

**Warm up questions:** *Aim\ make participants comfortable*

1. Please introduce yourself?

2. How old are you?

3. What is your education level?

4. What is your job?

5. How many children do you have?

6. How long is it since your wife’s pregnancy termination?

7. What happened to you when you found out that the fetus had a severe anomaly and had to be aborted? Please explain more?

**Interview guide questions in individual interviews with men whose wives have experienced pregnancy termination due to fetal abnormalities**

1. How do you think a man can support his wife from the time of the diagnosis of anomalies to the termination of pregnancy and afterward? Please explain?

2. Do you think the presence of a man in the delivery department at the time of miscarriage is necessary? Please explain more?

3. What expectations did your wife have of you after termination of the pregnancy? Please explain?
