# Supplementary material for: Supportive needs of women who have experienced pregnancy termination due to fetal abnormalities: a qualitative study from the perspective of women, men and healthcare providers in Iran
Source: BMC Public Health. 2019 May 3;19:507. doi: 10.1186/s12889-019-6851-9 (PMC6500064; doi:10.1186/s12889-019-6851-9)
Supplement: Supplementary file 3 — Interview guide during the face-to-face interviews with health care providers (midwives, nurses, gynaecologists, forensic medicine specialists, reproductive health specialists and psychologists) for the study conducted to determine the supportive needs of these women from the perspective of women, men and healthcare providers in Rasht Town, Iran, 2017–2018 (See methods section for further description). (DOCX 15 kb) [file 12889_2019_6851_MOESM3_ESM.docx]

**Additional file 3:** Interview guide during the face-to-face interviews with health care providers (midwives, nurses, gynaecologists, forensic medicine specialists, reproductive health specialists and psychologists) for the study conducted to determine the supportive needs of these women from the perspective of women, men and healthcare providers in Rasht Town, Iran, 2017-2018 (See methods section for further description).

**Introduction:** *Aim, to create appropriate atmosphere*

- Name of the interviewer and affiliation
- Purpose of the study
- Consent to take part in the study
- Confidentiality, explain how the data will be used
- Interview will last approximately 30-60 minutes
- Audio recorded to ensure interviewer can fully engage in the interview

**Warm up questions:** *Aim\ make participants comfortable*

1. Please introduce yourself?

2. How old are you?

3. What is your education level?

4. What is your job?

5. What is your work experience?

6. What experiences have you had with women who decide to end pregnancy after a diagnosis of fetal abnormalities during your years of activity? Please explain.

**Interview guide questions in individual interviews with health care providers**

1. What can health care providers do to meet the needs and improve the conditions for these mothers from diagnosis of abnormalities to miscarriage, discharge time, and even afterwards?

2. How do you think the health care providers can support women who have aborted their fetus due to abnormalities?
